# Brand first? The effect of hotel online word-of-mouth on consumer brand sensitivity

**DOI:** 10.3389/fpsyg.2022.986620

**Published:** 2022-08-18

**Authors:** Xianchun Li, Yiying Fan, Xin Zhong, Jiajing Hu

**Affiliations:** ^1^School of Business Administration, Faculty of Business Administration, Southwestern University of Finance and Economics, Chengdu, China; ^2^Sichuan Academy of Social Sciences, Chengdu, China; ^3^Business and Tourism School, Sichuan Agricultural University, Chengdu, China

**Keywords:** brand sensitivity, online word-of-mouth, reverse matching, functional value, symbolic value

## Abstract

With the e-commerce development and changing of hotels’ booking channels, the online word-of-mouth, as a new signal of quality, is becoming to attract more attention of consumers. Using the scenario experiment, this study explores the effect of online word-of-mouth on brand sensitivity of consumers during the decision making for hotel booking. The results show that if the information about hotels obtained is limited in the decision-making process, consumers would have a higher sensitivity to the hotel brand. Increasing information about the online word-of-mouth can effectively reduce consumers’ brand sensitivity to hotels. Besides, the moderating effect of the hotel grade on the relationship between the online word-of-mouth and brand sensitivity is affected by the scale of the negative differences of word-of-mouth.

## Introduction

Hotel industry is traditionally service-oriented, with great differences between products. Also, people need to travel to strange places for consumption. Compared to tangible products, it is more difficult for consumers to make accurate judgments about hotel service quality at the hotel reservation process due to their intangibleness (Baek and Ok, 2017; Yang et al., 2018; Hu et al., 2020). Especially through traditional offline channels, consumers have limited access to hotel choices and information of each one, such as hotel location, star level, brand and price, etc. Only the star level and brand can reflect the quality of hotel products (Lyu et al., 2017). However, the hotel star level cannot totally represent the level of quality, because hotel star indexes are too broad and there maybe have some differences between different hotels with the same star. Comparing with the hotel star level, as a commitment to quality, the brand’s indication for quality is more effective (O’neill and Mattila, 2010; Sürücü et al., 2019; Wang et al., 2019). Thus, when booking through traditional offline channels, consumers think high of hotels’ brand. This phenomenon that the brand is valued in the decision-making process is called Brand Sensitivity (Kapférer and Laurent, 1992). Because of consumers’ brand sensitivity, hotel enterprises must value their own brand construction, and invest a huge amount of brand marketing funds to strive to build brand image, spread brand value and enhance brand awareness every year (Lee et al., 2017; Sürücü et al., 2019). Meanwhile, in order to cater to consumers’ brand preference, traditional hotel distribution channels will also incline limited channel resources to well-known brand hotels (Lyu et al., 2017; Lam and Law, 2019). Thus, brand is extremely important for both hotel companies and consumers.

However, with the rapid development of hotel e-commerce and the profound change of hotel booking channels, more and more consumers are switching from traditional channels to online platforms to book hotels (Card et al., 2003; Park et al., 2007; Ku and Fan, 2009; Lam and Law, 2019). In China, booking volume from online platforms is more than 30% of the total sales volume of the hotel (Lyu et al., 2017), and this proportion is still rising. That means online platforms such as online travel agency (OTA) are becoming the main hotel sales channel (Ling et al., 2011; Chang et al., 2019; Lv et al., 2020), which can accommodate far larger number of hotels than traditional channels (Ku and Fan, 2009), and can provide more sales opportunities for non-brand hotels (Ling et al., 2011). For example, current famous OTAs in the worldwide, such as Ctrip (the biggest OTA in China) and Expedia, have shown numerous non-brand hotels. In addition, online platforms also allow consumers to post online reviews, share their personal experiences and evaluation of hotels, and generate online word-of-mouth (Ku and Fan, 2009; Litvin and Dowling, 2016). As a new quality signal, online word-of-mouth can provide decision-making suggestions for potential consumers (Godes et al., 2005; Yang et al., 2018; Yen and Tang, 2018). Recently research findings showed that, the effect of online word-of-mouth grows rapidly and it works more gradually when the quality role of brand has not yet been fully played (Yang et al., 2018; Yen and Tang, 2018). When booking a hotel through online platforms, consumers can obtain both the information of brand and online word-of-mouth. To this effect, it is necessary to figure out whether consumers still value brands? Will the importance of the brand be smaller than that in the past? Answers to these questions are essential to hotel companies’ brand stratagem.

In recent two decades, studies in hospitality industry have focused heavily on discussing and examining the overwhelming role of hotel brand for hotel companies and consumers (e.g., O’neill and Mattila, 2010; Xie and Heung, 2012; So et al., 2013; Litvin et al., 2016; Casidy et al., 2018). Previous literature has shown that since a strong brand can help to simplify consumers’ decision-making process by reducing perceived risks and increasing expectations, hotel consumers highly rely on hotel brand when making reservation (Mattila, 2007; Ku and Fan, 2009; Litvin et al., 2016; Casidy et al., 2018). In other words, brand sensitivity widely exists in consumer’s purchase decision making. However, some scholars have noticed that consumers’ brand sensitivity is not static, and changes with the change of decision-making environment (Wu et al., 2011). For example, Brown et al. (2012) found that in the development of traditional grocery stores to large supermarkets and then to online shopping platforms, consumers’ brand sensitivity changes with the change of shopping environment. Regarding the purchase situation (Brown et al., 2012), product category (or product characteristics) (Cassia et al., 2017; Casidy et al., 2018) and consumer involvement (e.g., Korai, 2017), consumers may also have different levels of brand sensitivity. Currently, hotel booking channels have evolved to online platforms, online word-of-mouth serving as a new quality clue has changed purchase situation, and consumers may not pay much attention to the brand. However, very little research has explored and verified whether the brand sensitivity in the hotel booking decision making process will be reduced. To fill in this gap, this paper aims to (1) examine the effect of hotels’ online word-of-mouth on consumers’ brand sensitivity; (2) verify hotel grade’s moderating role to the level of brand sensitive.

## Literature reviews and theoretical hypotheses

### The role of brand in the hotel reservation decision

The American Marketing Association (AMA) defines a brand as a name, sign, symbol, design, or a combination of them which is intended to identify the goods or services of one seller or a group of sellers and to differentiate them from those of competitors. Brand has become an important factor in the consumer decision because the brand can effectively reduce the perceived risk of consumers in the purchasing process (Chaudhuri and Holbrook, 2001; O’neill and Mattila, 2010; Casidy et al., 2018) have classified consumer perceived risk as functional and affective. A brand offers a mixture of functional, symbolic and emotional experiential benefits to satisfy the needs of consumers (Cheng, 2014). With the help of the brand, the quality of the purchased products can be guaranteed and then reduce consumers’ functional risks. At the same time, consumers can achieve satisfaction by purchasing brand products with symbolic functions and Sentimental functions, which can reduce the affective risks caused by the purchase of wrong products (O’neill and Mattila, 2010; Kladou et al., 2017; Casidy et al., 2018; Sürücü et al., 2019).

As a kind of intangible and service-type products, the quality evaluation of hotel products is more difficult than tangible products (Lam and Law, 2019). For the hotel reservation, consumers need to make decisions in a “time and space isolation” state. In time, consumers make consumptions after reservations; in space, consumers make reservations locally and then need to travel to different places for consumption, which further increase the decision-making risks (Baek and Ok, 2017; Yang et al., 2018; Hu et al., 2020). In this case, consumers need to seek effective means of evaluating the quality of hotel products to reduce the decision-making risks (Chang et al., 2019). In the traditional offline reservation channels, the limited clues of products quality which consumers can rely on mainly come from the hotel star and brand (Hu et al., 2019). However, the hotel star is only used to identify hotel grades. Due to budget constraints, consumers rarely conduct cross-star product comparisons in a single decision. In addition, there may still be a large quality gap between different hotels with the same star. Therefore, the hotel star is difficult to fully reflect the quality of hotel products today (Lyu et al., 2017). Then, in the purchase decision of the hotel products, the brand becomes the important identification that helps consumers identify the hotels’ quality (Kayaman and Arasli, 2007; Kladou et al., 2017; Casidy et al., 2018; Sürücü et al., 2019), and then the important factor for consumers’ hotels decisions.

### Brand sensitivity in the consumer decision making

In a period of time, the quality and value proposition of brand are relatively stable (Cheng, 2014; Lee et al., 2017). Even in different regions, its quality is guaranteed. Therefore, it makes the brand with quality promises and symbolism valued by consumers (Cheng, 2014; Casidy et al., 2018; Sürücü et al., 2019), which becomes an important clue for consumers to evaluate product quality and plays a decisive role in the decision making. Kapférer and Laurent (1992) defined the phenomenon that the brand is valued in the decision-making process as Brand Sensitivity. Since only by the concept of brand sensitivity can the true brand loyalty be distinguished from lazy repurchases (Odin et al., 2001; Imtiaz et al., 2019), the brand sensitivity of consumers has been proved to be a great significant variable for shaping of brand (Cheng, 2014). Hence, it is the foundation of the brand loyalty and the embodiment of the brand value. Brand sensitivity is a psychological construct that refers to the buyer’s decision-making process (Korai, 2017), only when the consumers are sensitive to brands will they form true attitude and behavior loyalty of brand and reduce the sensitivity to price (Casidy et al., 2018), resulting in higher premium and brand equity (Brown et al., 2012). Once consumers are no longer sensitive to brands, marketers’ investment on the brand will be lost. Given the importance of brand sensitivity, enterprises’ branding activities are meaningful.

However, the brand sensitivity of consumers is not changeless, it often changes with the decision-making environment changing (Degeratu et al., 2000; Wu et al., 2011; Cassia et al., 2017). On the one hand, researchers have generally found that the reference value of brands is higher when consumers obtain less information (Ku and Fan, 2009). That is, in the absence of product information, consumers will rely on the brand with a higher brand sensitivity under the halo effect (Richardson et al., 1994). On the other hand, under certain conditions, consumers are no longer dependent on the brand. A typical case is that when buying a commodity in large supermarkets, consumers may consider choosing the private brand of retailers rather than the manufacturer brand (Muratore, 2003; Mathews-Lefebvre and Valette-Florence, 2014), even though some private brands are far less famous than manufacturer brands. From the perspective of consumer behavior, the possible reason is that when the purchase environment changes, the level of consumers’ brand sensitivity decreases significantly. Existing research has revealed that factors such as price, brand quality differences (Wu et al., 2011), the competitive intensity (Brown et al., 2012), the importance and complexity of purchases (Brown et al., 2012; Casidy et al., 2018) and consumer involvement (Muratore, 2003; Mathews-Lefebvre and Valette-Florence, 2014; Korai, 2017) significantly influence brand sensitivity.

When booking hotels through traditional offline channels, consumers are undoubtedly sensitive to brands. They choose the well-known chain brand hotels (hereinafter referred to as “brand hotels”) more than non-reputable brand hotels and individual hotels (here in after referred to as “non-brand hotels”) (Ku and Fan, 2009). However, when the booking channels of hotel change, the information environment for consumer decision making also changes profoundly. Hotel products information in the online channel is very rich, and especially the word-of-mouth from consumers provides them a new product quality clue (Litvin and Dowling, 2016; Yang et al., 2018; Yen and Tang, 2018). Then, with the changing of decision-making environments, will the hotel brand sensitivity of consumers change? The existing researches on brand sensitivity haven’t a definitive answer, and the relationship between the word of mouth and brand remains to be explored deeply. If the word-of-mouth information reduces the consumers’ reliance on the brand, it weakens the competitive advantages of brand hotels, and brings more sales opportunities for non-brand hotels. Therefore, hotel marketers, especially non-brand hotel marketers, need to understand the effect of word-of-mouth on hotel brands. Marketers can better adapt online sales channels only if they understand the characteristics and internal mechanisms of consumer behaviors deeply.

### The weakening effect of hotels’ online word-of-mouth on consumers’ brand sensitivity

Since the emergence of online word of mouth, the communication mode of word-of-mouth has undergone tremendous changes, and its effect on consumer decisions has received more and more attention (Yang et al., 2018). Existing researches have widely confirmed that the volume, valence, mean value, variance and other factor of word of mouth have a significant effect on the sales of various products such as books (Gong et al., 2012), movies (Liu, 2006), restaurants (Lu and Feng, 2009), and tourist destinations (Lyu et al., 2017). Some studies have also shown that online word-of-mouth has the same effect on the sales of hotel products (Ye et al., 2011). The following question is, consumers can obtain hotels’ online word-of-mouth and brand information simultaneously in the scenario of hotel online reservations. In this situation, little is known about whether online word-of-mouth affects or even replace brands to some extent?

In fact, brand and online word-of-mouth are both external information clues used by consumers to evaluate the quality of hotel products (Richardson et al., 1994; O’neill and Mattila, 2010; Rajavi et al., 2019), which have different effect mechanisms on quality marking: the brand is the symbol and label of product quality, and it comes from product manufacturers, representing manufacturers’ commitment (O’neill and Mattila, 2010; Casidy et al., 2018); otherwise, the online word-of-mouth is derived from consumers, who share their own experience after the end of consumption (Yang et al., 2018; Yen and Tang, 2018). Although there are differences on the source and the generative mechanism, the hotel brand and online word-of-mouth can effectively mark the quality of hotel products (Vermeulen and Seegers, 2009; Yang et al., 2018; Xu, 2019). Therefore, consumers can use any one of them to complete the evaluation of hotel products. There is a certain degree of substitutability between the two. When booking hotels online, consumers cannot only obtain the brand information, but also online word-of-mouth of hotels. Based on the information asymmetry, the lack of product quality information in decision making is the most important source of brand sensitivity for consumers (Xu, 2019). The newly increased information from online word-of-mouth will help solve this information asymmetry dilemma, thus reducing consumers’ reliance on the brand when they are making decisions, and ultimately weakening consumers’ brand sensitivity.

H_1_: Increasing online word-of-mouth information can significantly reduce the brand sensitivity of consumers.

In reality, well-known brands could not always have a better word-of-mouth (Lyu et al., 2017). According to search and alignment theory (Pham and Muthukrishnan, 2002), consumers who initially have positive proattitudinal information about the brand and then are faced with negative attitudinal information that challenges the initial impression on brand, tend to revise this impression into the direction of the challenging information (Lv et al., 2022). When there is a conflict between the hotel brand and online word-of-mouth, that is, a brand hotel’s evaluation score is lower than a non-brand hotel, which are in a state of reverse matching, the exposed disadvantage of online word-of-mouth will offset the brand’s advantages, and consumers’ trust on the brand will be reduced due to the lower online word-of-mouth score (Bambauer-Sachse and Mangold, 2011; Brzozowska-Woś and Schivinski, 2019) (the online word-of-mouth score here refers to the mean value of customers’ word-of-mouth rating on the hotel booking webpage). Then consumers will less depend on brands when they are making decisions. Moreover, the greater the negative difference in the word-of-mouth score between brand hotels and non-brand hotels, the more intense the conflict will be (Bambauer-Sachse and Mangold, 2011), and the worse consumers’ trust on brands will decline.

H_2_: Compared to the positive matching, consumers’ brand sensitivity is lower when the brand matches the online word of mouth reversely.

H_3_: When the brand and online word-of-mouth are reversely matched, the greater the negative difference in the word-of-mouth score between the brand hotels and non-brand hotels is, the lower the brand sensitivity of consumers will be.

### The moderating role of hotel grade

Product values include functional values, symbolic values and emotional value values (Nasution and Mavondo, 2008). Because different grade hotels have different business strategies, there is a difference between low-grade and high-grade hotels in the product values contributing to customer (Ren et al., 2016). Low-grade hotels focus on providing good value for the money by offering functional products and services, While high-grade hotels focus on providing customers additive pleasure and comfort with premium products and services (Nasution and Mavondo, 2008; Yang et al., 2021), their product value contains functional, symbolic and emotional values, and prefers the symbolic and emotional values (Xu, 2019). Previous researchers have discussed the significant differences between behaviors of consumers when consumers are booking different grades of hotels (e.g., Becerra et al., 2013; Heo and Hyun, 2015; Ren et al., 2016). Therefore, the moderating effect of hotel grade on consumers’ reservation decisions needs to be explored further. The hotel brand can reflect three kinds of values at the same time. That is, it not only can be the guarantee of functional value, but also fully embody the symbolic and emotional value. In contrast, the word of mouth, especially the word-of-mouth ratings as an overall quality clue (Kim et al., 2016; Xu, 2019), can plays a certain role of replacing the brand when consumers evaluate the functional value of products. However, it cannot affect the symbolic and emotional value of products (Heo and Hyun, 2015; Lyu et al., 2017), meaning that it cannot replace the symbolism and affection of brand. So, the effect of online word-of-mouth on brand sensitivity is moderated by the hotel grade. for the low-grade hotels dominated by the functional value, consumers’ brand sensitivity can be reduced effectively by providing sufficient information of online word-of-mouth (Levin et al., 2003); while for high-grade hotel dominated by the symbolic and emotional value, the substitution role of online word-of mouth on brand has disappeared (Lockshin et al., 2006; Bambauer-Sachse and Mangold, 2011). So, the effect of online word-of-mouth on brand sensitivity is not significant, and the brand sensitivity of consumers will not change significantly with the matching modes between brand and online word-of-mouth and with the increase on the negative scores of online word-of-mouth.

H_4_: The effect of online word-of-mouth on the brand sensitivity is moderated by the hotel grade.

H_4a_: For low-grade hotels, increasing information of online word-of-mouth will significantly reduce consumers’ brand sensitivity. For high-grade hotels, increasing information of online word-of-mouth will not change consumers’ brand sensitivity.

H_4b_: For high-grade hotels, whether the brand and online word of mouth match positively or reversely, consumers’ brand sensitivity between them will not be significantly different.

H_4c_: For high-grade hotels, when the brand and online word of mouth match reversely, the scale of negative scores of online word-of-mouth between brand hotels and non-brand hotels has no significant effect on consumers’ brand sensitivity.

## Scenario experiment study

This study adopts a situational experiment method to analyze the changes of consumer brand sensitivity under different circumstances and their selection decisions. A total of three experimental groups are designed. Experiment 1 (E1) simulates the traditional offline booking channel, only with the hotel star and brand information; Experiment 2 (E2) simulates the hotel online booking channel and adds word-of-mouth information based on E1; Experiment 3 (E3) and Experiment 4 (E4) further compare the differences in consumer behaviors between low-grade and high-grade hotels.

### Experiment 1: Brand sensitivity of consumers in hotel reservation decisions

#### Experiment design

Experiment 1 simulated the traditional offline booking channel and only provided information on a hotel’s grade, brand, and price for the participants. In the experiment, two hotels are selected as target hotels: one is a brand hotel. According to the “ranking of brand scale within China’s Economy Chain Hotels in 2019,” Hanting Hotel is selected; the other is a non-brand hotel, with its virtual name “Yayuan” similar to “Hanting” in semantics. Since the location of the hotel cannot be changed after determined, it is not a marketing element that can be controlled. Therefore, this study excludes the effect of location factor and requires the participants to “do not consider the location.” In addition, the experiment further controls the hotel’s price, that is, all are set to 200 yuan/night. The experimental task is set to “You are going to travel to a city, and when you make a travel plan, you have checked out two adjacent budget hotels in the city. Please look at the following alternative hotel information and make a choice.”

#### Participants selection and experiment process

The experiment recruits the participants on a paid basis. Participants are required to be familiar with the hotel’s reservations and have certain hotel check-in experience, but they aren’t members of Hanting Hotels to prevent the effect of membership system. As the experiment begins, the experimenters displayed the relevant information of the two hotels to the participants through the computer screen, respectively. In order to avoid the sequence effect, the display order of the two hotels has been exchanged. Afterward, participants are asked to make decisions and answer a questionnaire about brand sensitivity. The measurement of brand sensitivity we used includes six items in the form of five-point Likert scale (Lachance et al., 2003). Then the 5-level Likert scale is used to test the brand familiarity. The familiarity of Hanting Hotel being less than 4 points, and the familiarity of Yayuan Hotel being more than 2 points do not meet the experimental requirements and should be removed. In the end, each experimental group retains only the first 60 eligible samples.

#### The experiment results

**FIGURE 1:**
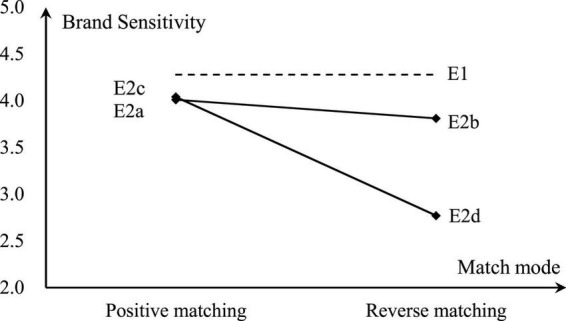
Effect of word-of-mouth on the brand sensitivity.

The Experiment Results show (Table 1) that 100% of the participants choose Hanting Hotel. The Cronbach’s α of the brand sensitivity scale is 0.82, indicating that the internal consistency of the scale is satisfactory and has a higher level of reliability. The brand sensitivity reaches 4.28 (dotted line in Figure 1), being at a higher level. In the sequence effect test, since the participants all choose Hanting Hotel, the differences in the participants’ decisions under different display orders are not significant. The one-way ANOVA test results also show that differences on the brand sensitivity are not significant (*t* =−0.68, *P* = 0.50), indicating that there is no sequence effect in the experiment. In summary, in the traditional channel situation, participants have a higher degree of brand sensitivity and completely prefer a brand hotel.

**TABLE 1:** The experiment results.

Experiments	Gender (%)	Age	Familiarity		Experiment time (S)	Brand sensitivity	Selection ratio of brand hotels	Sequence effect	Reliability
			
			Brand hotels	Non-brand hotels					
E1	62	38.8	4.43	1.28	68.0	4.28	1.00	−0.68/−	0.82
E2a	58	35.7	4.35	1.35	293.4	4.01	1.00	0.99/−	0.86
E2b	57	40.2	4.42	1.37	405.2	3.81	0.88	0.37/0.30	0.90
E2c	65	42.4	4.45	1.25	267.7	4.04	1.00	1.05/−	0.85
E2d	60	37.9	4.40	1.22	369.5	2.77	0.43	0.97/1.41	0.94
E3	66	41.4	4.37	1.35	57.9	4.51	1.00	−0.57/−	0.93
E4a	54	46.3	4.29	1.20	117.6	4.54	1.00	−1.15/−	0.91
E4b	68	39.1	4.43	1.29	220.6	4.39	0.95	0.96/0.53	0.86
E4c	62	43.2	4.41	1.34	343.2	2.88	0.53	0.26/−0.24	0.84

The value of gender column in the table is the male proportion in the experiment; the value of sequence effect column is t value of ANOVA test for brand sensitivity and decision result; the reliability is Cronbach’s α value.

### Experiment 2: Reducing effect of online word-of-mouth on brand sensitivity

#### Experiment process

Experiment 2 simulates the online booking channel and adds word-of-mouth information of hotels based on Experiment 1. Word-of-mouth scores are considered an indication of customer’s overall evaluation of hotel by many studies (e.g., Xu, 2019), so we add word-of-mouth scores as word-of-mouth information in Experiment. Before the experiment, we first collect the word-of-mouth ratings of 100 hotel stores in Beijing, with their brands ranking the top 10 of the domestic budget hotels through the largest OTA platform in China.^1^ The average value is calculated to be approximately 4.5 and the standard deviation is about 0.3. So, it can be approximated thought that this is the word-of-mouth level of brand budget hotels in Beijing. In the experiment, the word-of-mouth information is collected from two Hanting hotels in Beijing from Ctrip. The experiment collects the top 20 word-of-mouth from each of them and groups into α_1_ and α_2_. Among them, the average score of α_1_ is 4.5, which means the average level. The average value of α_2_ is 4.8, and the difference between the two is one standard deviation. The word-of-mouth information groups, α_1_ and α_2_, are assigned to two alternative hotels. Theoretically, there are two matching methods. Corresponding Experiment 2 is further subdivided into two sub-experiments: (1) positive matching. Hanting matches the word-of-mouth groupα_2_, and Yayuan matches the word-of-mouth group α_1_in Experiment 2a. There is a positive difference in word-of-mouth scores between the brand hotel and non-brand one; (2) reverse matching. In the Experiment 2b, Hanting matches α_1_ and Yayuan matches α_2_. At this time, there is a negative difference between the brand hotel and non-brand one. The Experiment Process is the same as that of Experiment 1, and each experimental group retains the first 60 valid samples.

#### Experiment results

The results of the sequence effect test of Experiment 2a and Experiment 2b are not significant as shown in Table 1. In Experiment 2a, all the participants still choose Hanting Hotel, but the brand sensitivity decreases. The *t*-test result of independent sample is mean difference *Δm* =−0.27, *t* = 2.26, and *P* = 0.03. These show that consumers’ consideration of word-of-mouth information during decision-making reduces the importance of the brand. Therefore, H_1_ has been supported.

Compared with the positive matching of Experiment 2a, the backstage timing in Experiment 2b shows that the reverse matching between word of mouth and the brand leads to a significant extension of the decision-making time for participants, and the selection ratio for the brand hotel is significantly reduced to 0.88. The brand sensitivity is also further reduced to 3.81. Although the decrease in brand sensitivity is not significant compared to Experiment 2a, it is significantly lower than the level in Experiment 1 (*Δm* = −0.47, *t* = 3.65, *P* = 0.00). When the level of word of mouth is in conflict with the quality of the brand, participants deepen their thinking about word of mouth. However, due to the insignificant difference in the test results of brand sensitivity, H_2_ has not yet been supported.

#### Supplementary experiments

The difference in word-of-mouth scores between Experiment 2a and Experiment 2b is only one standard deviation. Considering that the difference in the word-of-mouth score may affect the Experiment Results (Brown et al., 2011), this study adds two groups of experiments with larger differences in word-of-mouth scores (Experiment 2c and Experiment 2d). First, a group of word-of-mouth information with a lower score α_3_ is supplemented with an average score of 4.2, which differs from α_2_ by two standard deviations. Experiment 2c adopts a positive matching method of Hanting matching the word-of-mouth group α_2_ and Yayuan matching the word-of-mouth group α_3_. In Experiment 2d, the Hanting matching the word-of-mouth group α_3_ and Yayuan matching the word-of-mouth group α_2_ are used in the reverse matching method. Then we continue to conduct more experiments. In the results of Experiment 2c, the selection ratio of Hanting is still 100%, and the degree of brand sensitivity for participants is not significantly different from that of Experiment 2a (*Δm* = 0.03, *t* =−0.27, *P* = 0.79). That is, when the word-of-mouth information is consistent with the brand information, the increase of positive difference in the word-of-mouth score does not significantly change the brand sensitivity of consumers. However, in the results of Experiment 2d, Hanting’s selection ratio is only 0.43 (Δ*m* =−0.57, *t* =−8.78, *P* = 0.00). This selection ratio and the degree of brand sensitivity for the participants (Δ*m* =−1.27, *t* =−7.54, *P* = 0.00) are all significantly lower than those of Experiment 2c. The conclusions of Experiment 2a and Experiment 2b comprehensively indicate that H_2_ can only be supported when there is a larger difference in word of mouth scores. Comparing with Experiment 2b, in Experiment 2d, Hanting’s selection ratio (*Δm* = −0.45, *t* = −5.49, *P* = 0.00) and brand sensitivity (*Δm* = −1.04, *t* = −5.85, *P* = 0.00) have also significant declines, that is, when the word-of-mouth information is inconsistent with the brand information, the increase of reverse difference in the word-of-mouth scores has increased consumers’ consideration of word-of-mouth and the importance of brand has been further weakened correspondingly. Therefore, H_3_ has been supported.

The results of Experiment 1 and Experiment 2 are presented in Figure 1.

### Experiment 3 and experiment 4: Moderating role of hotel grade

#### Experimental materials

Both Experiment 1 and Experiment 2 use the budget hotel. Experiments 3 and Experiment 4 make the changes at the hotel level to take luxury hotels into account. In the experiment, Hilton Hotel is chosen as the brand hotel, while the virtual hotel is still adopted for the non-brand hotel. “Paris,” the name of Hilton Hotel’s heir, Hilton Paris, is chosen as the virtual hotel’s name. In the experiment, participants are told that the two hotels are five-star ones and the prices are controlled at 1,000 yuan/night. The experimental procedure is the same as before. In the sample acquisition, through the paid sample service from a questionnaire survey company, we select groups who have stayed in five-star hotels, and the income level of the samples is controlled at a monthly income of 10,000 yuan or more.

#### Process and results of experiment 3

In Experiment 3, referring to Experiment 1, only hotels were replaced by high-star ones. The results of participants’ decision-making are the same as before: 100% participants select brand hotels, and the brand sensitivity reaches the highest of 4.51 in all experimental groups, which is significantly higher than that in experiment 1 (Δm = 0.23, *t* = −2.78, *P* = 0.01). That is, in the traditional offline decision-making environment where word-of-mouth information is lacking, the hotel grade plays a positive moderating role on the brand sensitivity of participants.

#### Process and results of experiment 4

Experiment 4a and Experiment 4b refer to Experiment 2a and Experiment 2b, respectively. The word-of-mouth information of the two hotels is still taken from Ctrip.com. Actually, they are two Hilton hotels in Beijing, which are grouped into β1 and β2, respectively. The average word-of-mouth scores are also 4.5 and 4.8.

Experiment 4a takes a positive matching between the brand and word-of-mouth, that is, the Hilton brand is paired with the word-of-mouth group of β2, and the Paris brand is paired with the word-of-mouth group of β1, and then the experiment is conducted. In the Experiment Results, the brand sensitivity of participants still maintains the high level as Experiment 3 (Δm = 0.03, *t* = 0.218, *P* = 0.28), and the brand hotel’s selection ratio remains at 100%. Increasing the positive matching word-of-mouth do not significantly affect the brand sensitivity and decision-making.

Experiment 4b takes into account the reverse matching of the brand and word-of-mouth. Although the Hilton Hotel’s word-of-mouth score is slightly lower, the number of participants who choose Hilton Hotel is still an absolute majority, reaching 0.95, being not significant in differences with Experiment 3 (Δm = −0.05, *t* = 1.78, *P* = 0.08) and Experiment 2b (Δm = 0.07, *t* = 1.78, *P* = 0.08), in Experiment Results. Participants continue to maintain a higher level of brand sensitivity, which is not significantly lower than that of in Experiment 3 (Δm = −0.12, *t* = 1.09, *P* = 0.28), but significantly higher than that of Experiment 2b (Δm = 0.58, *t* = −4.00, *P* = 0.00), indicating that even if the word-of-mouth information by the reverse matching is added, if the word-of-mouth score is at a high level, the word-of-mouth still cannot effectively affect the brand sensitivity and decision-making of participants.

Referring to Experiment 2d, we further expand the negative differences of the word-of-mouth scores and establish a word-of-mouth score group β3 with an average of 4.2. Since the result of positive matching is more obvious, it should be consistent with Experiment 3 and Experiment 4a. Then, the Experiment 4c only needs to consider the reverse matching, that is, the word-of-mouth group of β3 is paired with the Hilton brand, and the Paris brand is still paired with the word-of-mouth group of β2, and then experiment is conducted again. At this time, the selection ratio of brand hotels is significantly lower than that of Experiment 3 (Δm = −0.47, *t* = 7.19, *P* = 0.00), which is at the same level as Experiment 2d (Δm = 0.10, *t* = 1.09, *P* = 0.28); similarly, the brand sensitivity is also significantly lower than that of Experiment 3 (Δm = −1.63, *t* = 8.78, *P* = 0.00), reaching the same level as in Experiment 2d (Δm = 0.11, *t* = 0.47, *P* = 0.64). That is, when the word-of-mouth score is at a low level, the word-of-mouth by the reverse matching significantly affects the brand sensitivity and decision-making of participants.

Experiment 4c shows that even for high-grade hotels, increasing word-of-mouth information may change the brand sensitivity of consumers, that is, H4a fails to pass the test. Besides, there is a significant difference between the reverse matching and positive matching, that is, H4b also fails the test. According to the results of Experiment 4b and Experiment 4c, the scale of negative differences of online word-of-mouth between brand and non-brand hotels has a significant effect on consumers’ brand sensitivity. So, H4c fails to pass the test. The reason why H4a, H4b, and H4c could not pass the test is the moderating effect of the hotel grade on the relationship between word-of-mouth information and brand sensibility, and it is also affected by the scale of the negative differences of word-of-mouth. Therefore, H4 has not be supported.

**FIGURE 2:**
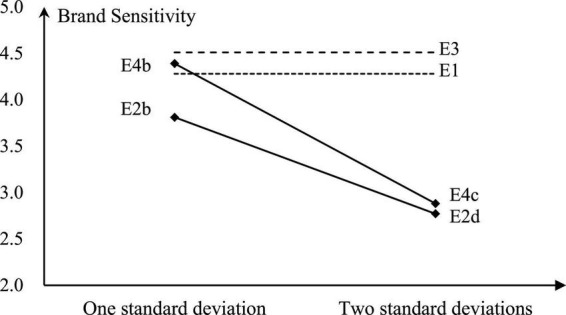
The moderating role of hotel grade.

The results of Experiment 3 and Experiment 4 are presented in Figure 2 showing the moderating role of hotel grade.

## Conclusion and marketing suggestions

### Conclusion

This study analyzes the variation of brand sensitivity and its antecedent factors in the hotel reservation decision of consumers through four consequential experiments, and draws the following conclusions:

(1) When making decisions, if consumers can only get limited information of hotels, they show a higher degree of brand sensitivity. When booking hotels through traditional offline channels, there is very little information for consumers that can be used to assess the quality of hotels. At this time, the brand plays an important role and becomes hotels’ quality signal that consumers mainly concern (O’neill and Mattila, 2010; Casidy et al., 2018). In Experiment 1, it is prominent that the brand sensitivity of participants is at a higher level.
(2) Increasing quantity of word-of-mouth information can effectively reduce consumers’ brand sensitivity to hotels. With the addition of word-of-mouth information, the brand sensitivity of consumers when they are making decisions will decline significantly. Compared with offline channels, through the online channels consumers can obtain word-of-mouth information from other users (Yen and Tang, 2018; Yang et al., 2018). The means of assessing the quality of hotel products for consumers has become rich, and the degree of dependence on brands has declined. In Experiment 2a and Experiment 2c, it can be seen that the addition of word-of-mouth does reduce the brand sensitivity of participants. While in Experiment 2b, it further changes participants’ decisions. A considerable number of participants start to choose non-brand hotels. In the Experiment 2d, where the score differences of word-of-mouth increase, this selection ratio continues to increase.
(3) The moderating role of hotel grade on the relationship between word-of-mouth information and brand sensitivity is also affected by word-of-mouth levels. For low-grade hotels that emphasize on functional value, word-of-mouth, as a clue of hotel products’ quality, can play a better substitution role for brands that play the same role (Bambauer-Sachse and Mangold, 2011; Lockshin et al., 2006). However, in high-grade hotels with symbolic value, the relationship between word-of-mouth and brand has become more complicated. When word-of-mouth scores of brand hotels and non-brand hotels are all at a high level, word-of-mouth information do not affect the brand sensitivity of participants significantly (Experiment 4a, Experiment 4b). The main reason is that, for high-grade hotels, consumers are mainly pursuing the symbolic value and emotional value, while the brand’s symbolic value cannot be replaced by word-of-mouth, which is consistent with the previous deduction of H2. At this time, even if the non-brand hotel’s word-of-mouth score is slightly higher, consumers will still tend to choose a brand hotel. Obviously, the role of the brand is greater than word-of-mouth. However, when the word-of-mouth score of a high-star hotel is relatively low (Experiment 4c), participants’ brand sensitivity declines sharply. The reason for this phenomenon is that the functional value, symbolic value and emotional value of hotel products are not completely juxtaposed, and the functional value is the basis of symbolic value and emotional value (Nasution and Mavondo, 2008). Although high-grade hotels embody the symbolic value mainly, their symbolic value only makes sense when their functional value is guaranteed. Therefore, when the brand hotels’ word-of-mouth scores are too low, participants will give priority to the quality information delivered from the word-of-mouth (Bambauer-Sachse and Mangold, 2011; Brzozowska-Woś and Schivinski, 2019), then the brand sensitivity reduces, and participants prefer to the non-brand hotels. At this time, the role of word-of-mouth is greater than the brand.

### Marketing implications

The change of consumer decision-making information environment affects the brand sensitivity and the ultimate choice of consumers in making decisions. The quality-revealing function of brand is declining and the function of word-of-mouth is growing. Therefore, with the increasing development of online channels, hotel marketers should pay more attention to the power of word of mouth.

For brand hotels, they need to be aware of the challenges brought about by online booking channels. Brand is not the only decisive factor. Previous marketing strategies that emphasized brand building must be properly adjusted to give more attention to online word-of-mouth. In the marketing process, hotel marketers should strengthen the monitoring and management of word-of-mouth, avoid negative word-of-mouth to offset the brand advantage, and actively establish the word-of-mouth consistently with the brand, thereby exerting the superposition effect of word-of-mouth and brand.

For non-brand hotels, online channels bring more opportunities for them. Brand building often requires a higher marketing investment, which is very difficult for non-brand hotels with limited funds. But the cost of building a good word-of-mouth is much smaller, it is easier to reach, and it can effectively make up disadvantages for the brand. Therefore, non-brand hotels, especially budget hotels, should emphasize the effect of word-of-mouth and pay attention to the accumulation of word-of-mouth.

For online booking platforms such as OTA, the significant effect of word-of-mouth information from these platforms on consumer decisions may increase the importance of them. The online booking platforms can expand the cooperation with hotels and reflect the diversified value of platforms by helping hotels develop word-of-mouth management.

## Research limitations and prospects

This study explores the changes in consumers’ brand sensitivity under different decision-making scenarios and analyzes the effect of word-of-mouth information on consumers’ brand sensitivity and the moderating role of hotel grade. Restricted by research conditions and periodization, this study mainly considers the effect of word-of-mouth information on brand sensitivity. In fact, compared to offline booking channels, OTA provides more information, including not only word-of-mouth information but also official information such as photos, words, and other types of information. Future researches can further analyze the increase in the total supply of information and the possible effect of different types of information. In addition, this study only considers the ideal situation of the two hotels. When the number of alternative hotels increases, whether consumers’ behaviors would be affected require further explorations.

## Data availability statement

The original contributions presented in this study are included in the article/supplementary material, further inquiries can be directed to the corresponding author/s.

## Author contributions

XL: conceptualization, methodology, formal analysis, investigation, data curation, and writing—original draft. YF: writing—original draft, review and editing, formal analysis, methodology, and data curation. XZ: formal analysis, methodology, data curation, and review and editing. JH: conceptualization, methodology, writing—original draft, review and editing, supervision, funding acquisition, and project administration. All authors contributed to the article and approved the submitted version.

## Footnotes

1
www.ctrip.com
